# Reduction of mitomycin C is catalysed by human recombinant NRH:quinone oxidoreductase 2 using reduced nicotinamide adenine dinucleotide as an electron donating co-factor

**DOI:** 10.1038/sj.bjc.6603414

**Published:** 2006-10-10

**Authors:** D Jamieson, A T Y Tung, R J Knox, A V Boddy

**Affiliations:** 1 Northern Institute for Cancer Research, University of Newcastle upon Tyne, Paul O'Gorman Building, Medical School, Newcastle upon Tyne NE2 4HH, UK; 2 Protherics plc., Building 115, Porton Down Science Park, Salisbury, Wiltshire SP4 0JQ, UK

**Keywords:** mitomycin C, NQO1, NQO2, bioreductive drugs

## Abstract

NRH:Quinone Oxidoreductase 2 (NQO2) has been described as having no enzymatic activity with nicotinamide adenine dinucleotide (NADH) or NADPH as electron donating cosubstrates. Mitomycin C (MMC) is both a substrate for and a mechanistic inhibitor of the NQO2 homologue NQO1. NRH:quinone oxidoreductase 2 catalysed the reduction of MMC at pH 5.8 with NADH as a co-factor. This reaction results in species that inhibit the NQO2-mediated metabolism of CB1954. In addition, MMC caused an increase in DNA cross-links in a cell line transfected to overexpress NQO2 to an extent comparable to that observed with an isogenic NQO1-expressing cell line. These data indicate that NQO2 may contribute to the metabolism of MMC to cytotoxic species.

Mitomycin c (MMC) is an anticancer antibiotic used in the treatment of superficial bladder cancer and other tumours. As a prodrug, the parent compound is relatively nontoxic compared to the highly electrophilic metabolites formed following sequential or simultaneous two electron reduction of the quinone to the hydroquinone (Iyer and Szybalski, 1963). Many enzymes have been implicated in the catalysis of MMC including NQO1 (Siegel *et al*, 1992) and interest in NQO1-directed cancer chemotherapy arose following the observation that NQO1 is more highly expressed in tumour tissue when compared with matched healthy tissue, for example lung (Schlager and Powis, 1990).

Evidence supporting NQO1 as a candidate enzyme in the reduction of MMC has been reported. Isogenic cell line models transformed into a high NQO1 phenotype are more sensitive than parental cell lines to MMC (Mikami *et al*, 1996). A correlation between MMC GI_50_ and NQO1 activity in the National Cancer Institute 60 cell line panel presented evidence of a more heterogeneous nature that NQO1 has a role in MMC bio-activation (Fitzsimmons *et al*, 1996). Although MMC has been demonstrated to be a substrate for NQO1, a large amount of enzyme and an acidic pH are needed to see a reaction, indicating that MMC is a poor substrate for NQO1 (Siegel *et al*, 1992). An added level of complexity is that, when activated, MMC alkylates NQO1, irreversibly inhibiting the enzyme (Siegel *et al*, 1993). Other studies have failed to identify an association between sensitivity to MMC and NQO1 activity (Robertson *et al*, 1992) and other enzymes have been implicated in the bioreductive activation of MMC including NADPH Cytochrome *C* reductase (Pan *et al*, 1984) and nicotinamide adenine dinucleotide (NADH) cytochrome *b*_5_ reductase (Hodnick and Sartorelli, 1993). However, the one electron reductases implicated in MMC activation are unlikely to be responsible for NQO1 independent activation of MMC in tumours in an aerobic environment.

NRH:quinone oxidoreductase 2 (NQO2) has recently been described as an enzyme of surprises and mysteries (Vella *et al*, 2005). One of the mysteries assumed of NQO2, in contrast to the widely studied homologue NQO1, is that it can use neither NADH nor NADPH as an electron donor. Instead, NQO2 requires the non-biogenic compound NRH to facilitate enzymatic activity (Knox and Chen, 2004). In this study, the ability of human recombinant NQO2 to reduce MMC utilising NADH as an electron donor was characterised, under conditions similar to those used to characterise NQO1 catalysed reduction of MMC. The inhibition of enzyme activity by the NQO2 inhibitor quercetin and the NQO1 inhibitor dicoumarol was also investigated. The effect of hrNQO2 bioreductive activation of MMC on NQO2 activity, as measured by CB1954 reductase activity, was determined. An increased alkylation of DNA in V79 chinese hamster lung fibroblasts transformed to overexpress either NQO1 or NQO2 was also observed, compared to a plasmid transformed control.

## MATERIALS AND METHODS

hrNQO1, hrNQO2 (prepared as described in Wu *et al* (1997)), bovine serum albumin (BSA), lactose, Tris-HCL, MMC, phosphate dibasic, phosphate monobasic, deduced NADH, dicoumarol, quercetin and ammonium formate were purchased from Sigma-Aldrich (Poole, Dorset, UK). Phosphate-buffered saline (PBS) was purchased from Invitrogen (Paisley, UK) HPLC grade acetonitrile and methanol were purchased from Fischer Scientific (Loughborough, UK). CB1954 and NRH were obtained from Protherics PLC (Cheshire, UK), NQO1 and NQO2 transformed V79 Chinese hamster lung fibroblast cells were also supplied by Protherics PLC and were generated as described previously (Knox *et al*, 2000), Genesis C18 120A 4 *μ*m 100 × 4.6 mm HPLC column was purchased from Jones chromatography (Pontypridd, UK).

Reduction of MMC was carried out in a 100 mM potassium phosphate buffer at 25°C at either pH 5.8, 6.5, or 7.4 for 30 min. Initial concentrations were 100 *μ*M MMC, 4 mM NADH and 162 nM hrNQO1 or 162 nM hrNQO2 or 4.2 *μ*g/ml of BSA with lactose and PBS buffer salts. Inhibition studies were carried out at pH 5.8 with the addition of either 100 *μ*M quercetin or 100 *μ*M dicoumarol. At time 0 and 30 min, 150 *μ*l aliquots from the reaction mixture were mixed with 450 *μ*l acetonitrile, vortex mixed and centrifuged at 12 000 **g** for 5 min. Supernatant (400 *μ*l) was evaporated under nitrogen and the residue resuspended in 400 *μ*l water. Isocratic HPLC separation of MMC was carried out on a Waters (Elstree, UK) Alliance 2695 separation module and 2487 dual wavelength detector with detection at 365 nm. Mobile Phase comprised of 72% 10 mM ammonium formate (pH 6.5) : 28% acetonitrile at a flow rate of 0.3 ml min^−1^. Injection volume was 5 *μ*l with a run time of 10 min.

NQO2 kinetic studies were carried out as above at pH 5.8 with a range of NADH concentrations of 0.25–4 mM and 100 *μ*M MMC. Aliquots were removed at 0, 5, 10, 15, 20 and 30 min for HPLC analysis. Mitomycin C concentrations remaining were interpolated from a standard curve and initial rates were calculated as the initial slope of a second-order curve of the data.

### NQO1 activity assay

The NQO1 activity of cell lysis supernatants was determined by an adaptation of a previously published method (Gan *et al*, 2001). The reaction monitored the reduction of DCIP with and without dicumarol and the NQO1 activity was taken as the dicumarol-sensitive fraction. Cell lysate was added to a solution containing final concentrations of 0.04 mM DCIP and ±0.01 mM dicumarol in 25 mM Tris-HCL buffer (pH 7.4). The reaction was initiated by the addition of NADH to a final concentration of 0.2 mM and a final volume of 300 *μ*l. The absorbance at 600 nm was measured at 10 s intervals over 2.5 min at room temperature, monitored using a Spectramax 250 spectrophotometer. All reactions were carried out in 96-well plates and each sample was measured in triplicate. The concentration of oxidised DCIP remaining at each time point was calculated from a molar extinction coefficient of 21 ml *μ*mol^−1^cm^−1^. The reaction rate in nanomolar DCIP reduced per min per mg of total protein was calculated from a plot of *A*_600_ against time.

### NRH:quinone oxidoreductase 2 activity assay

CB1954 reduction was assayed as previously described (Knox *et al*, 2000) with minor modifications. V79 lysate (for determination of cellular NQO2 activity) or an aliquot of hrNQO2 following incubation of +/− MMC and +/− NADH (for determination of non-competitive inhibition of NQO2 by MMC) were added to a borosilicate tube containing NRH and CB1954 in PBS to give final concentrations of 500 *μ*M NRH and 100 *μ*M CB1954 in a volume of 1 ml at a pH of 7.4. Reactions were incubated at 37°C and 90 *μ*l aliquots were removed at 0, 5, 10, 20, 30, 40, 60 and 80 min, added to 90 *μ*l acetonitrile, vortex mixed and centrifuged at 12 000 **g** for 5 min. The CB1954 concentration remaining at each time point was determined by HPLC analysis and interpolated from a standard curve. Isocratic HPLC separation of CB1954 was carried out on a Waters Alliance 2695 separation module and 2487 dual wavelength detector with detection at 325 nm. Reaction mixture (15 *μ*l) : acetonitrile (50 : 50) was injected onto a Genesis *C*_18_ 120A 4 *μ*m 100 × 4.6 mm HPLC column with a mobile phase of 80% 0.02 M phosphate pH 6 : 20% methanol at an isocratic flow of 1 ml min^−1^. CB1954 was detected at a wavelength of 325 nM and a retention time of 7 min and the concentration remaining was interpolated from a standard curve. The rate of CB1954 reductase activity was calculated by the initial velocity per mg of protein.

DNA alkylation was assessed by modified comet assay as previously described (McKenna *et al*, 2003). Exponentially growing V79 cells transformed to over express either NQO1 (hdt), NQO2 (TH7) or transformed with empty plasmid (f179) were incubated with 10 *μ*M MMC in a final volume of 1 ml for 1 h at 37°C in separate eppendorfs. Following treatment, cells were centrifuged at 1200 **g** for 3 min and washed twice with PBS, mixed 1/10 with LM aragrose and 75 *μ*l aliquots placed on Trevigen comet assay slides (Gaithersburg, MD, USA) slide. Once the agarose had set, the slides were irradiated at 10 Gy prior to lysis and subsequent denaturation at pH 13. Cells underwent horizontal electrophoresis at a constant 300 mA and between 15 and 20 V for 20 min. The slides were washed in deionised H_2_O and fixed in 70% EtOH and allowed to dry overnight. The cells were stained with SYBR green and 3D images acquired by confocal microscopy. Comets were analysed by Komet 5 software (Wirral, UK)

## RESULTS

### Reduction of mitomycin C catalysed by hrNQO1 and hrNQO2

**Figure 1:**
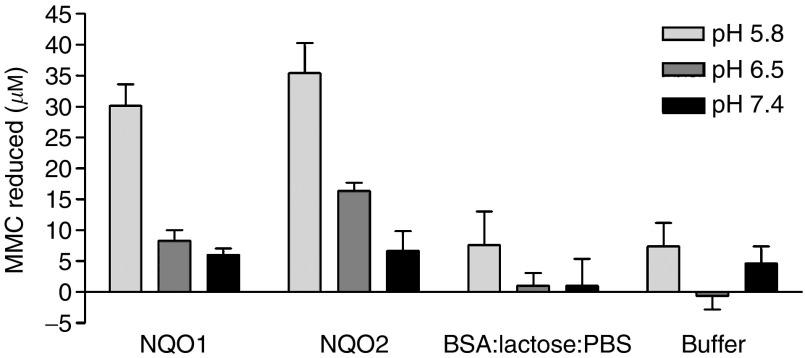
Reduction of MMC catalysed by hrNQO1 and hrNQO2 compared with BSA : lactose : PBS and no protein controls. At pH 5.8 the mean MMC reduced in the hrNQO1 reaction (30±3.4 *μ*M) and the hrNQO2 (35±4.9 *μ*M) reaction were significantly less than the MMC reduced in the no protein control (*P*=0.002 and 0.002, respectively, *t*-test). Reactions were carried out at pH indicated and room temperature for 30 min using 4 mM NADH, 100 *μ*M MMC. Data are mean and s.e. of 5 (pH 5.8) or three independent experiments.

Either hrNQO1 or hrNQO2 alone catalysed the reduction of 100 *μ*M MMC by 30±3 and 35±5 *μ*M, respectively, over 30 min incubation at room temperature and pH 5.8, with NADH as an electron donor (*P*=0.002 and 0.002, respectively, when compared to buffer, *t*-test). No reduction of MMC was seen in a solution containing BSA, lactose and PBS salts under the same conditions (Figure 1). Reduction of MMC was also catalysed by both hrNQO1 and hrNQO2 enzymes at pH 6.5, but by neither enzyme at pH 7.4 (Figure 1). NRH:quinone Oxidoreductase 2 activity was dependent on NADH concentration, with the rate being approximately 60% of maximum at the lowest NADH concentration tested (250 *μ*M) (data not shown). No reduction of MMC occurred at any pH with either enzyme in the absence of NADH (data not shown).

### Inhibition of NRH:quinone oxidoreductase 2 catalysed reduction of mitomycin C

**Figure 2:**
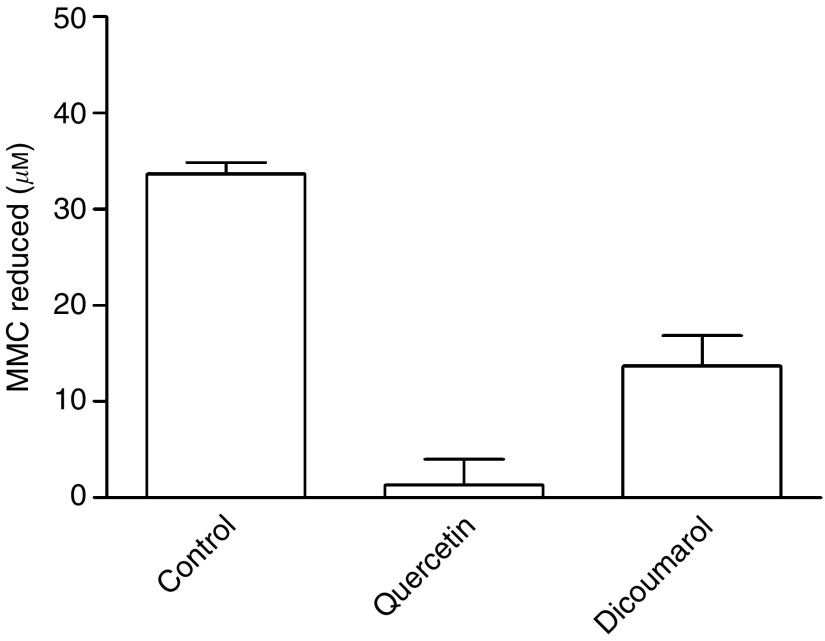
Reduction of MMC catalysed by hrNQO2±quercetin or dicoumarol. Incubation with 100 *μ*M quercetin and hrNQO2 resulted in inhibition of reduction of MMC (*P*=0.002 compared with no inhibitor). Incubation with 100 *μ*M dicoumarol and hrNQO2 resulted in partial inhibition of reduction of MMC (*P*=0.017 compared with no inhibitor). Reactions were carried out at pH 5.8, room temperature for 30 min using 100 *μ*M MMC and 4 mM NADH. Data are mean and s.e. of three independent experiments.

The reduction of MMC catalysed by hrNQO2 was completely inhibited by 100 *μ*M quercetin and partially inhibited by 100 *μ*M dicoumarol when compared to the reaction in the absence of the inhibitor (Figure 2).

### Autodestructive mitomycin C catalysis by NRH:quinone oxidoreductase 2

**Figure 3:**
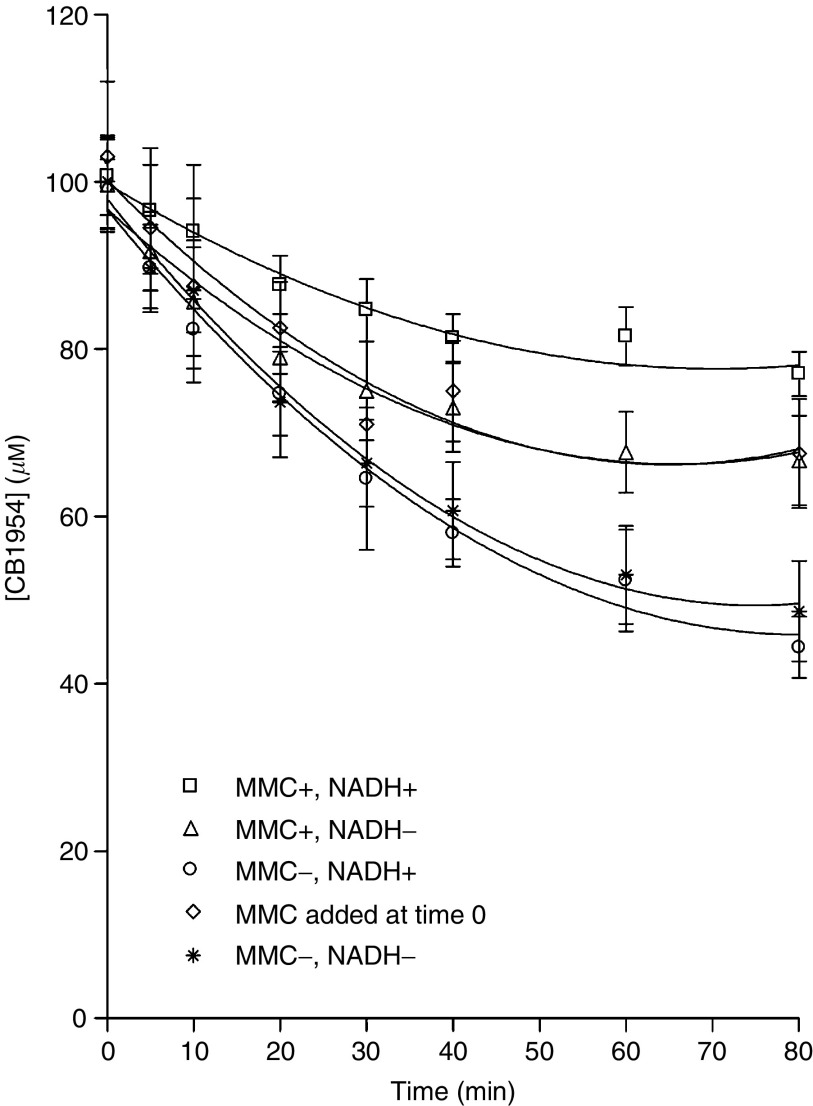
Reduction of CB1954 at 37°C, pH 6.3 using NRH as an electron donor and 200 *μ*l aliquots of a 30 min preincubation of 16.2 nM hrNQO2 with or without MMC or NADH as described in the text. MMC added (rhombi) represents the addition of MMC at time 0 of the CB1954 reaction (difference between MMC+NADH+ and MMC+NADH−, *P*=0.0033, 2 Way ANOVA). Lines are second-order curves used to calculate initial velocity. Data are the mean and s.e. of three independent experiments.

NRH:quinone oxidoreductase 2 activity, as measured by reduction of CB1954 in the presence of NRH, was inhibited by 29±2% following an incubation of hrNQO2 with MMC in the absence of NADH (pH 5.8, room temperature for 30 min) (Figure 3), when compared to a 30 min preincubation of hrNQO2 without MMC. Preincubation with MMC and NADH resulted in a greater degree of inhibition (50±1% of control).

### Comet assay on transformed V79 cells

**Figure 4:**
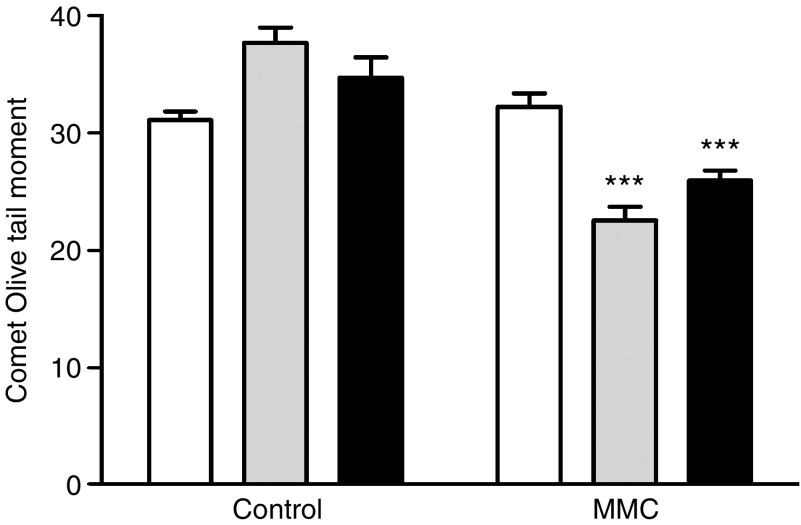
Comet olive tail moment of plasmid (F179, white), NQO2 (TH7, grey) or NQO1 (hdt, black) transformed cells following incubation with or without 10 *μ*M MMC. Data represent the mean and s.e. from one of three independent experiments. ^***^=*P*-value of <0.0001, *t*-test of mean compared with untreated control.

V79 chinese hamster lung fibroblasts cells transformed to overexpress NQO1 or NQO2 were used to determine the relative degree of DNA damage caused my MMC. Neither NQO1 nor NQO2 activity was detectable in the empty plasmid transformed cell line (F179). NRH:quinone oxidoreductase 2 was undetectable in the NQO1 transformed cell line (hdt) which had a mean NQO1 activity of 1487±29 nmol min^−1^ mg^−1^. NQO1 was undetectable in the NQO2 transformed cell line (TH7) which had a mean NQO2 activity of 8.2±2.9 nmol min^−1^ mg^−1^. Following a 1 h incubation of each cell line in 10 *μ*M MMC at physiological pH and 37°C the mean Olive tail moment was unchanged compared to untreated cells in the F179 cells. In contrast both the NQO1 (hdt) and NQO2 (TH7) cell lines had a decreased Olive tail moment following MMC treatment compared with untreated cells (Figure 4).

## DISCUSSION

NRH:quinone oxidoreductase 2 catalyses the reduction of MMC utilising NADH as an electron donor by a mechanism that is blocked by the known NQO2 inhibitor quercetin. This reduction was shown to generate reactive metabolites, which inhibited the CB1954 reductase activity of NQO2 to a greater degree than inactivated MMC. Also, there was a lower comet olive tail moment in NQO2 expressing cells treated with MMC compared with cells with no detectable NQO2 activity, again indicating the generation of DNA-reactive species. This observation is contrary to the perceived lack of enzymatic function of NQO2, but is not inconsistent with the literature. Nicotinamide adenine dinucleotide is used to elute hrNQO2 during affinity chromatography with cibacron blue as a ligand and hrNQO2 has a *K*_m_ for NADH three- to four-fold higher than that of NQO1 (Wu *et al*, 1997). The 43 carboxy terminal residues of NQO1 which interact with the ADP section of NADH do not totally restore NADH binding or activity to NQO2 (Wu *et al*, 1997) and additional amino-acid differences in NQO2 compared with NQO1 in the catalytic site are believed to contribute to the difference in affinity. It has been suggested that the function of his161 in reduction of NQO1 by NADH prior to substrate reduction can be achieved by asn161 in NQO2 (Bianchet *et al*, 2004).

Despite the low affinity and very low activity of NQO2 for NADH, human recombinant NQO2 has been reported to have a low enzymatic activity when high concentrations of NADH are used as a co-factor and menadione as a substrate (Wu *et al*, 1997). As a result of the low affinity of NQO2 for NADH, with most substrates the concentration of NADH will be the rate-limiting factor. However, MMC is a poor substrate for NQO1 and substrate concentration may be the rate-limiting factor for reduction of MMC catalysed by either NQO1 or NQO2. The original observation of MMC reduction catalysed by NQO1 used 5.2 *μ*g/ml (169 nM) purified rat hepatic NQO1 (Siegel *et al*, 1992) and this is comparable with the 162 nM hrNQO2 or hrNQO1 used in the present study. Despite the nonphysiological conditions used in the cell-free systems described here, NQO2 expression in an isogenic cell model also resulted in more DNA alkylation, as measured by the comet assay, than was seen in an NQO1 and NQO2 null background.

Many enzymes have been implicated in the bioreductive activation of MMC including NQO1, cytochrome *C* reductase, cytochrome *b*5 reductase, xanthine oxidase and xanthine dehydrogenase (reviewed by Seow *et al* (2004)). However, of these enzymes, only xanthine dehydrogenase functions as a two electron reductase, the others catalysing a one electron reduction of the MMC quinone to a semiquinone. In an aerobic environment semiquinone is spontaneously oxidised to the parent compound with the production of free radicals. While this is potentially damaging, it is not believed to be the primary mechanism by which MMC exerts toxicity. An MMC reductase activity, immunologically related to NQO1 was identified in NQO1 −/− mice and may be partially attributable to NQO2 (Joseph *et al*, 1996; Joseph and Jaiswal, 2000). This activity was inhibited by NQO1 antibodies and dicoumarol and the published experiment used 1 mM NADH, a level that in the current study was sufficient to catalyse reduction of MMC (data not shown). A further study suggested this activity was due to *M*_r_ 58 000 glucose regulatory protein (GRP58) (Celli and Jaiswal, 2003). There is no published observation that mouse GRP58 is immunologically related to NQO1 or that human GRP58 catalyses the reduction of MMC. While this paper was in preparation it was reported that, in the absence of NRH, kerotinocytes from the NQO2−/− mouse are less sensitive to MMC than keratinocytes from wild-type mice (Celli *et al*, 2006). It was also observed that CHO cells transformed to overexpress NQO2 were more sensitive to MMC than wild-type cells and that recombinant mouse NQO2 with NRH can catalyse the reduction of MMC with subsequent generation of DNA alkylating species at pH 5.8 *in vitro* (Celli *et al*, 2006). These observations are supportive of the data presented here, but raise the question of whether endogenous electron donors for NQO2 exist. The NQO2 transformed V79 cell lines used in this study are sensitive to the NQO2 substrate CB1954 only in the presence of added NRH (Knox *et al*, 2000), indicating the absence of any endogenous NQO2 co-factor in this system.

The activity described in this study is unlikely to be extrapolated to other quinone substrates of NQO1 given the unique combination of low affinity of NQO1 for MMC and the low affinity of NQO2 for MMC and NADH. Mitomycin C is clearly metabolised by NQO2 in the presence of NADH to reactive species that both inhibit NQO2 and react with DNA to form cross-links. This study offers no suggestion that NQO2 functions physiologically as an enzyme. However, these data indicate that NQO2 may contribute to the bioreductive activation of MMC, particularly in an aerobic environment.
